# Divergent chemotactic sensing in *Acanthamoeba* reveals ligand-promiscuous, threshold-tuned pattern recognition without canonical formyl peptide receptors

**DOI:** 10.1093/femsml/uqag023

**Published:** 2026-06-27

**Authors:** Viktor Hermaraj, Brendan W Wren, Fauzy Nasher

**Affiliations:** Department of Infection Biology, London School of Hygiene and Tropical Medicine, Keppel St, London WC1E 7HT, United Kingdom; Department of Infection Biology, London School of Hygiene and Tropical Medicine, Keppel St, London WC1E 7HT, United Kingdom; Department of Infection Biology, London School of Hygiene and Tropical Medicine, Keppel St, London WC1E 7HT, United Kingdom

**Keywords:** *Acanthamoeba*, chemotaxis, MAMPs, PRRs, Glycans, GPCRs

## Abstract

Chemotaxis, the directed movement of an organism towards nutrients or away from noxious agents is a fundamental process for the survival of many micro-organisms. We combined high-resolution imaging, microfluidic gradients, and frame-by-frame tracking to re-evaluate *Acanthamoeba* chemotaxis to microbial glycans [mannan, mannose, *N*-acetyl-d-glucosamine (GlcNAc), *N*-acetyl-muramic acid (MurNAc)] and peptides [*N*-formyl methionyl-leucyl-phenylalanine (fMLP) and Boc-Phe-Leu-Phe-Leu-Phe (BOC-FLFLF)]. Our quantitative tracking results on *Acanthamoeba castellanii* confirm the core patterns in the original studies reported by Schuster and Levandowsky; attraction to fMLP and GlcNAc and lack of response to MurNAc or the peptide antagonist BOC-FLFLF, while revealing previously missed attraction to mannan. In contrast, *Acanthamoeba polyphaga* demonstrated a more restricted response, with significant chemotaxis observed only toward fMLP, and lack of motility in the presence of MurNAc or BOC-FLFLF. Notably, formyl peptide responses were differentially modulated: BOC-FLFLF reduced fMLP-induced directionality in *A. castellanii* without impairing motility, while in *A. polyphaga*, it suppressed both velocity and orientation. When considered alongside genomic analyses that do not reveal a canonical metazoan-like formyl peptide receptor, these behavioural differences suggest that formyl peptide sensing in *Acanthamoeba* relies on a divergent, pattern-recognition–like signalling strategy, rather than a conserved FPR homolog. These distinct chemoattractant “signatures” are consistent with micro-niche adaptation, and we hypothesise that fine scale tuning of receptor thresholds to local prey spectra contributes to the observed differences between the tested strains. By revisiting classical paradigms, this study offers new perspectives on *Acanthamoeba* chemotaxis and supports emerging models of protist pattern recognition paralleling innate immunity.

## Teaser

Conserved microbial glycans and peptides drive distinct chemotactic responses in *Acanthamoeba. Acanthamoeba castellanii* CCAP1501/10 responds to a broader range of ligands than *Acanthamoeba polyphaga* CCAP1501/15. Despite strong responses to the bacterial peptide fMLP, bioinformatic analyses failed to identify canonical metazoan-like formyl peptide receptors, suggesting the existence of a divergent, ligand-promiscuous sensing system underlying amoebal chemotaxis.

## Introduction

*Acanthamoeba* species,, including *Acanthamoeba castellanii* and *Acanthamoeba polyphaga*, are ubiquitous free-living protists that inhabit soil, water bodies, air, and human-made environments like swimming pools and plumbing systems (Rayamajhee et al. 2022). These organisms play vital ecological roles as bacterivores, shaping microbial communities and nutrient cycles (Mungroo et al. 2021, Price Christopher et al. 2024). They can be opportunistic pathogens capable of causing amoebic keratitis and granulomatous amoebic encephalitis, particularly in immunocompromised individuals or contact-lens wearers (Marciano-Cabral and Cabral 2003, Zhang et al. 2023).

Early work by (Schuster & Levandowsky 1996a) established the quantitative study of *A. castellanii* chemotaxis, demonstrating directed migration toward stimuli such as *N*-formyl-methionyl-leucyl-phenylalanine, Lipopolysaccharides (LPS), lipid A, lipoteichoic acid, cyclic AMP, and *N*-acetylglucosamine (GlcNAc), but not toward mannose, mannosylated BSA, or *N*-Acetylmuramic acid (MurNAc) and Boc-Phe-Leu-Phe-Leu-Phe (BOC-FLFLF) (Schuster and Levandowsky 1996a).

Subsequent investigations revealed the presence of high-affinity mannose-binding proteins (MBPs) on *A. castellanii*, which facilitate adhesion to mannose-rich ligands on host cells and microbes, enhancing phagocytosis and cytotoxicity (Marco Garate et al. 2006). Genomic and protein-level analyses have characterized varying mannose- and laminin-binding protein repertoires (MBP, MBP1, LBP) across different *Acanthamoeba* species and genotypes, suggesting evolutionary diversification of prey recognition systems (Clarke et al. 2013, Corsaro 2022, Matthey-Doret et al. 2022). The *A. castellanii* genome also encodes additional candidate recognition proteins, including D-galactoside/L-rhamnose-binding lectins (RBLs), peptidoglycan recognition proteins (PGRPs), and H-type lectin domain proteins (Clarke et al. 2013, Nasher and Wren 2024).

Recent work indicates that *A. castellanii* detects conserved microbe-associated molecular patterns (MAMPs), and that glycan presentation often governs recognition (Nasher and Wren 2023). In this context, the term “microbial glycans” refers to surface-exposed carbohydrate motifs on microbes, LPS inner cores/O-antigens, peptidoglycan (MurNAc–GlcNAc), capsular polysaccharides, β-1,3-glucans, and mannans/high-mannose N-glycans, which function as conserved MAMPs, as illustrated by studies in which *Escherichia coli* engineered to display only the Kdo₂–lipid A inner core were internalized at near wild-type levels. Preincubation with purified Kdo₂–lipid A, but not lipid A alone, blocked uptake, indicating that the two Kdo residues linked to lipid A are necessary and sufficient for recognition in this system (Liu and Koudelka 2023). Similarly, *A. castellanii* binds β-1,3-glucan on fungal cell walls, and masking these polymers diminishes uptake of Histoplasma (Ferreira Md et al. 2024). Uptake of *Campylobacter jejuni* depends on *O*-linked glycans on the major flagellin (FlaA); mutating the glycosylation sites abrogates this interaction (Nasher and Wren 2023). Together, these reports point to a common logic: *Acanthamoeba* recognizes glycan-decorated microbial surface assemblies—LPS inner cores, glucan-rich walls, and *O*-glycosylated flagella—where the conserved appendage is the scaffold and glycan presentation fine-tunes engagement. This pattern is consistent with a modular recognition system (lectin-like receptors for sugars alongside other sensors for peptides), paralleling PRR-based detection in multicellular hosts (Li and Wu 2021, Nasher and Wren 2024). At the molecular level, *Acanthamoeba* mannose-binding proteins and mammalian lectins (e.g. mannose receptor, collectins) show domain-level similarities and functional analogies, consistent with convergent evolution of microbial recognition pathways (Ferreira et al. 2022). Understanding chemotaxis in free-living amoebae matters ecologically, these protists structure bacterial communities, and evolutionarily, because their MAMP-guided navigation parallels PRR-based discrimination in innate immunity. By quantifying ligand-specific guidance in two species, we connect functional foraging strategies to conserved recognition logics that predate animals (Nasher and Wren 2024).

Although *Acanthamoeba* chemotaxis toward microbial ligands has been described, the mechanistic basis linking these behaviours to underlying receptor systems remains unresolved. In particular, whether peptide-driven chemotaxis reflects canonical metazoan-like receptor architectures or alternative sensing strategies has not been addressed.

Despite technological advances tracking microbes, comparative chemotaxis assays across *Acanthamoeba* species remain scarce. Prior studies have largely focused on *A. castellanii* (Kuburich et al. 2016, Schuster and Levandowsky 1996a), and quantitative chemotaxis of *A. polyphaga* towards microbial glycans has not been reported, even though lectin-mediated glycan recognition has been described for this species (Elloway et al. 2004). Here, we provide the first rigorous, quantitative comparison of *A. polyphaga* chemotaxis to this ligand panel under microfluidic gradients, using defined microbe-associated ligands and modern microfluidic imaging with frame-by-frame tracking. We use “chemotaxis” to include both attraction and chemo-repulsion; however, because a canonical chemorepellent for *Acanthamoeba* under microfluidic gradients has not been established, our assays focus on attraction metrics. We revisit classical chemotaxis assays using time-lapse imaging and quantitative cell tracking to analyse *A. castellanii* strain (CCAP1501/10) responses to mannan, mannose, GlcNAc, MurNAc, fMLP, and the antagonist BOC-FLFLF, selected as representative microbial-associated glycans and peptides previously implicated in amoebal recognition. Importantly, we also conduct a direct comparative chemotactic profiling of *A. polyphaga* (CCAP 1501/15), two well-characterised laboratory strains commonly used in experimental studies of amoebae biology, offering fresh insights into sugar sensing and lectin-mediated navigation.

Our comprehensive chemotaxis metrics, covering multiple microbial-associated ligands and peptide stimuli, provide a foundational framework for understanding prey discrimination by *Acanthamoeba* in environmental and pathogenic contexts. By applying modern live-tracking technology to classic assays, this work paves the way for exploring how MAMP-driven microbial recognition in protists parallels the evolution of innate immune discrimination in warm-blooded hosts.

## Methods

### Slide preparation and media composition

All experiments were conducted using Ibidi μ-Slide Chemotaxis chambers (Ibidi, Germany), which are specifically designed for live-cell imaging and chemotactic assays. Peptone–Yeast–Glucose (PYG) medium was freshly prepared and syringe-filtered (0.22 µm) for amoebal growth. For chemotaxis assays, glucose-free defined medium (ADM: NaCl: 1 g, KCl: 0.04 g, MgSO₄·7H₂O: 0.02 g, CaCl₂: 0.01 g, Na₂HPO₄: 0.14 g, KH₂PO₄: 0.06 g, FeCl₃: 0.5 mg at pH 7.5), which comprises only defined salts and iron. The omission of glucose and other nutrients ensured that observed amoeboid movement reflects ligand-driven migration rather than background metabolic stimuli.

### Amoeba culture and preparation

*Acanthamoeba castellanii* CCAP1501/10 and *A. polyphaga* CCAP1501/15 [*Culture collection of Algae and protozoa* (*Scottish Marine Institute*)] were cultured axenically in PYG medium at 25°C under standard atmospheric conditions in 75 cm² Corning™ tissue culture flasks containing 25 ml medium. Initial cultures were incubated for four days to reach log-phase growth. Amoebae were harvested at a concentration of approximately 2.5 × 10⁶ cells/ml, as determined by manual counting using a haemocytometer.

Prior to each experimental session, cultures were gently dislodged from flask surfaces by tapping the flasks against the workbench. The cell suspension were centrifuged at 4000 × g for 2 min. The supernatant was discarded, and the amoeba pellet was resuspended in 25 ml ADM. Cell dispersal was facilitated by pipetting and vortex mixing to ensure homogeneous distribution.

### Slide inoculation and chemotactic assay setup

Ibidi μ-Slides were loaded using the manufacturer’s protocol. Briefly, the central channel was inoculated with amoeba suspensions of either *A. castellanii* or *A. polyphaga*. One chamber reservoir well always contained ADM alone, while the right well was loaded with ADM supplemented with test stimulus at 1 mM concentration. Stimuli used include mannan, mannose, *N*-acetylglucosamine (GlcNAc), *N*-Acetylmuramic acid (MurNAc), *N*-formyl-methionyl-leucyl-phenylalanine (fMLP), and the antagonist Boc-Phe-Leu-Phe-Leu-Phe (BOC-FLFLF). Mannan, due to its higher potency, was used at 10–20 nM.

Ligand concentrations were selected based on previously published studies of *Acanthamoeba* chemotaxis (Schuster and Levandowsky ) and were further refined through preliminary experiments to ensure robust and reproducible responses within the microfluidic gradient system.

### Live-cell imaging

Live-cell imaging was performed using a Zeiss confocal microscope (LSM880) controlled via ZEN software. Time-lapse recordings were acquired over 2500 frames at a rate of one frame every 2.5 s (total duration ∼104 min) at objective 10x. Raw imaging data were exported from ZEN as uncompressed AVI files for subsequent analysis.

### Image processing and cell tracking

Time-lapse image analysis was conducted using Fiji (a distribution of ImageJ) with pre-installed plugins. For quantitative tracking, a Substack was generated with the input range set from frames 1–2000 at 25-frame intervals (i.e. every 62.5 s). This yielded an 80-frame AVI suitable for chemotaxis analysis. If amoeba motility was delayed, Substack frame ranges were adjusted (e.g. 101–2100–25, 301–2300–25) to capture periods of active migration.

Cell tracking was performed via the *Manual Tracking*. Between 15–20 individual cells were tracked per AVI. Tracking data were saved as .txt files compatible with the Ibidi Chemotaxis and Migration Tool software.

### Chemotactic analysis

Tracking files were imported into the Ibidi Chemotaxis and Migration Tool. Settings for analysis were as follows: Slices used: 1–80, X/Y calibration: 1.662 µm per pixel, Time interval: 62.5 s. X/Y calibration was determined from ZEN field-of-view data, with 851 µm mapped across 512 pixels, yielding 1.662 µm/pixel. All data were processed and interpreted according to Ibidi’s analysis guidelines. All tracking data are included in Supplementary File_1 and Supplementary File_2, for *A. castellanii* and *A. polyphaga*, respectively.

We omitted the forward migration index (FMI), the projection of a trajectory onto the gradient axis divided by path length, ranging from −1 to +1, widely used to quantify chemotactic bias. In our μ-Slide setup, cells were seeded in the central channel, ADM was placed in the left reservoir, and chemoattractant in the right reservoir. Because the central channel is open to both reservoirs, the solute gradient relaxes continuously as the attractant diffuses across the channel and into the opposite reservoir. Without concurrent gradient tracing (e.g. a fluorescent tracer), the gradient direction and magnitude are time-varying, and may even invert late in the run. Since FMI assumes a fixed, known gradient axis, using it here would bias values toward zero (or spuriously negative) as the gradient flattens/changes, making FMI unreliable for inference. We therefore focused on gradient-agnostic metrics—velocity, directness (net/accumulated distance), and accumulated distance, benchmarked against ADM controls and supported by trajectory plots.

### Identification of putative formyl peptide receptors in *Acanthamoeba*

To assess whether chemotactic responses to N-formyl peptides are mediated by a canonical formyl peptide receptor (FPR), we performed a systematic bioinformatic interrogation of *Acanthamoeba* genomes and proteomes. Analyses were conducted using the curated *A. castellanii* Neff genome (GCA_000313135.1) and the currently available *A. polyphaga* genome assembly (GCA_000826345.1). Formal BUSCO completeness metrics were not reported for the original genome assemblies used in this study. The *A. castellanii* Neff assembly was selected because it represents the most extensively curated and widely used reference genome for *Acanthamoeba*, whereas the currently available *A. polyphaga* assembly remains less comprehensively annotated. Genome assemblies were selected based on availability and annotation quality as reference datasets and do not correspond directly to the experimental strains used in this study. Analyses were therefore interpreted as genome-informed rather than strain-resolved. Protein datasets were obtained from publicly available genome annotations (NCBI RefSeq) associated with each assembly, and no *de novo* gene prediction or proteome reconstruction was performed.

Predicted proteomes were screened using HMMER (hmmscan v3.3) against the Pfam-A database to identify candidate G protein–coupled receptors (GPCRs). Searches focused on Pfam families associated with peptide-sensing GPCRs, including rhodopsin-like receptors (PF00001; Family 1), secretin-like receptors (PF00002; Family 2), Dictyostelid cAMP receptors (PF08395; Family 5), and Frizzled/Smoothened receptors (Family 6). To reduce false-positive matches arising from large multidomain proteins, analyses were restricted to proteins between 250 and 900 amino acids in length, and domain-level E-value thresholds of ≤1 × 10⁻¹⁰ were applied.

Candidate GPCR-like sequences were further evaluated by BLASTP against the NCBI non-redundant protein database to assess homology and domain context. Transmembrane topology was predicted using TMHMM (v2.0) and Phobius (Käll et al. 2004), and sequences were manually inspected for conserved Class A GPCR motifs, including the DRY/DRH motif in transmembrane helix 3, the CWxP motif in helix 6, and the NPxxY motif in helix 7. Proteins exhibiting fragmented transmembrane predictions, excessive length, or homology to cytosolic kinases, adaptor proteins, transporters, or RNA-associated factors were excluded from consideration as bona fide GPCRs. Across both genomes, PF00001-annotated candidates identified by HMMER were predominantly assigned to non-receptor protein classes based on BLASTP annotation and lacked a coherent seven-transmembrane topology, supporting their exclusion from canonical GPCR classification.

Putative GPCR candidates identified in *A. castellanii* were cross compared with orthologous sequences in *A. polyphaga* where possible, acknowledging the incomplete *A. polyphaga* genome assembly. Given the more complete annotation and curation of the *A. castellanii* reference genome, detailed candidate curation and reporting were prioritised for this species, while *A. polyphaga* analyses were used comparatively to assess conservation of candidate features under the same filtering criteria. A complete list of all candidate sequences, RefSeq accession identifiers, annotations, and classification criteria is provided in Supplementary File_4 to ensure transparency and reproducibility.

### Statistics

All quantitative data are reported as mean ± standard deviation (SD), with each condition replicated in three biological replicates. Statistical differences between treatment groups and the ADM (control) were evaluated using Two-way ANOVA followed by Dunnett’s multiple comparisons test, which controls the family-wise error rate when comparing multiple treatments against a single control. A two-tailed *P*-value <0.05 was considered statistically significant.

## Results and discussion

### Chemotactic responses of *A. castellanii* and *A. polyphaga* to microbial sugars and peptides

**Figure 1:**
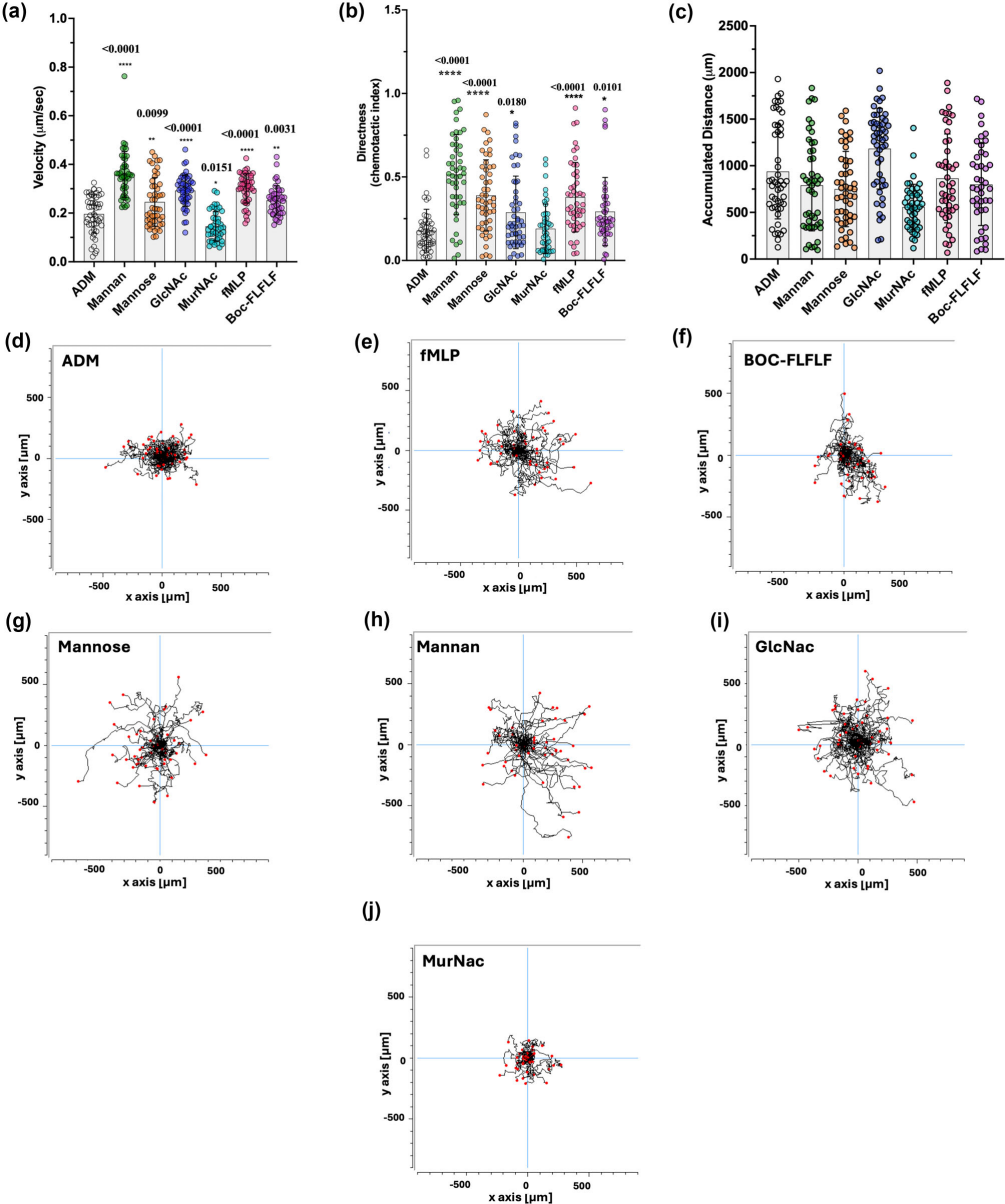
Chemotactic responses of *A. castellanii* to microbial ligands. Quantification of migration (a) velocity, (b) directness, and (c) accumulated distance in the presence of ADM (control), mannan, mannose, GlcNAc, MurNAc, fMLP, and BOC-FLFLF. Data represent means ± SD from three biological replicates. Asterisks indicate statistically significant differences relative to ADM (*P < 0.05, **P < 0.01, ****P < 0.0001).

**Figure 2:**
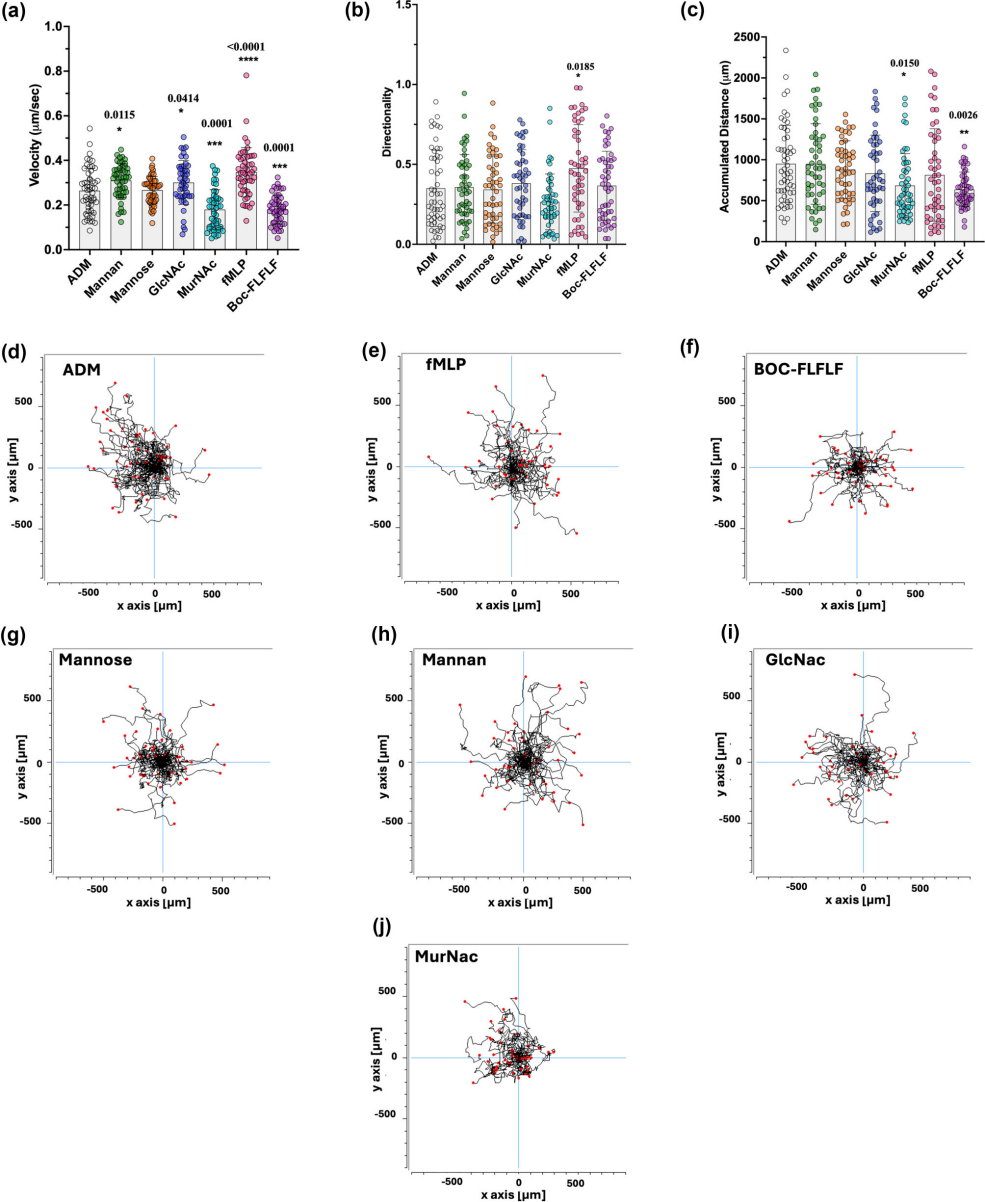
Chemotactic responses of *A. polyphaga* to microbial ligands. Quantification of migration (a) velocity, (b) directness, and (c) accumulated distance in the presence of ADM (control), mannan, mannose, GlcNAc, MurNAc, fMLP, and BOC-FLFLF. Data represent means ± SD from three biological replicates. Asterisks indicate statistically significant differences relative to ADM (*P < 0.05, **P < 0.01, ****P < 0.0001; Dunnett’s test).

To revisit and expand upon early investigations into amoeboid chemotaxis (Schuster and Levandowsky 1996a) we evaluated the responses of *Acanthamoeba castellanii* and *A. polyphaga* to a panel of microbial surface-associated sugars (mannan, mannose, GlcNAc, and MurNAc), the bacterial peptide fMLP, and its analogue BOC-FLFLF. Using ibidi μ-Slide chemotaxis chambers and manual tracking across 2000 frames (2.5 s/frame), we analysed ∼40–50 cells per condition over three biological replicates. Tracking data were processed using the ibidi Chemotaxis & Migration Tool to generate trajectory maps and quantitative metrics of velocity, directness, and accumulated distance (Figs. 1 and 2).

Relative to the ADM baseline, *A. castellanii* showed significantly increased velocities in response to mannan (*P* < 0.0001), mannose (*P* = 0.0164), GlcNAc (*P* < 0.0001) and fMLP (*P* < 0.0001). Interestingly, BOC-FLFLF also produced a modest but significant increase (*P* < 0.0059), while MurNAc showed statistically significantly lower velocity (*P* < 0.0230). These findings highlight a preferential chemokinetic response toward mannose-rich glycans and bacterial peptides (Fig. 1a). Similarly, significant increases in directional persistence were observed for mannan, mannose, GlcNAc, fMLP, and BOC-FLFLF. Notably, mannan induced the highest directional bias (*P* < 0.0001). The stronger response to mannan relative to mannose is consistent with an avidity requirement, whereby multivalent mannose displays (e.g. mannan) cluster mannose-reactive lectins (e.g. MBP) and amplify downstream signalling, whereas monomeric mannose affords low-avidity engagement and weak directional bias (Casillo et al. 2021). At the nanomolar concentrations used, bulk viscosity is unlikely to account for the rightward bias; instead, mannan’s slower diffusion likely sustains a steeper, longer-lived gradient near the source, and limited surface adsorption could introduce a mild haptotactic component. Together with multivalent lectin engagement, these physical and biochemical factors plausibly underlie the stronger directional response relative to small sugars or fMLP.

MurNAc failed to enhance directionality significantly, suggesting these molecules may be insufficient cues for directional migration on their own (Fig. 1b). Unlike velocity and directness, accumulated distance did not significantly differ across conditions, suggesting that overall path length was not as sensitive an indicator of chemotactic response in this assay format. A borderline effect was noted with GlcNAc (*P* < 0.0503), which may merit further exploration under longer timeframes or with automated tracking (Fig. 1c). Trajectory plots revealed pronounced directional migration toward fMLP, mannan, and GlcNAc, while tracks for ADM appeared more isotropic. MurNAc and BOC-FLFLF produced reduced path lengths (Fig. 1d–j).

By contrast, *A. polyphaga* (CCAP150/15) exhibited a more selective response profile (Fig. 2). Significantly increased velocities were observed only for mannan (*P* < 0.0115), GlcNAc (*P* < 0.0414), and fMLP (*P* < 0.0001), with fMLP producing the most pronounced effect. Directional persistence was significantly enhanced solely by fMLP (*P* < 0.0185), while other cues—including mannose and GlcNAc—failed to elicit directional migration. Accumulated distance was significantly reduced in response to MurNAc (*P* < 0.0150) and BOC-FLFLF (*P* < 0.0026), suggesting inhibitory effects on sustained migration unique to this species (Fig. 2a–c). Trajectory plots show that *A. polyphaga* exhibits directional migration only toward fMLP, while responses to mannan, mannose, and GlcNAc appear non-directed, and MurNAc and BOC-FLFLF visibly suppress motility—highlighting a restricted chemotactic repertoire relative to *A. castellanii (*Fig. 2d–j).

Comparative analysis of the tested strains reveals that *A. castellanii* (CCAP150/10) displays a broader and more robust chemotactic repertoire, responding to multiple glycans and peptides with increased speed and directional bias, whereas *A. polyphaga* (CCAP150/15) shows a more constrained profile with directional responses largely limited to fMLP and reduced long-range migration in the presence of certain ligands. These patterns show differences between the tested strains in receptor repertoires and signalling thresholds rather than a single shared pathway. For mannose-class cues specifically, the stronger response to mannan than mannose is consistent with multivalent engagement of a mannose-binding lectin; by contrast, responses to GlcNAc and to fMLP likely involve distinct lectin(s) and a peptide-sensing GPCR-like mechanism, respectively. Consistent with a multivalency model, pre-exposure of *A. castellanii* to free mannose yielded an “adhesion without detectable uptake” phenotype, which was further supported by gentamicin protection assays showing no recoverable intracellular *Campylobacter jejuni*_GFP_, consistent with inhibition of productive internalisation. This phenotype was compatible with competitive masking of mannose-sensitive binding sites (e.g. MBP or related lectins) and insufficient multivalent engagement to trigger uptake of the model bacterium *C. jejuni* (Supplementary File. 3  Figure S1). Baseline uptake of *C. jejuni* under identical conditions in the absence of mannose is shown in Supplementary File. 3 and Fig. 2b, where active internalisation is observed. *Campylobacter jejuni* was employed here as a representative Gram-negative bacterial prey organism to probe *Acanthamoeba* chemotactic sensing and uptake logic, with the focus placed on host behaviour and sensing strategy rather than pathogen-specific mechanisms. Unlike *E. coli*, which is commonly used as a general amoebae food source, *C. jejuni* exhibits defined, glycan-dependent interactions with *Acanthamoeba*, making it a suitable model for investigating ligand-specific recognition processes (Nasher and Wren 2023).

This working model does not imply that MBP alone mediates uptake; rather, parallel recognition routes and downstream signalling pathways are likely to operate in concert, potentially involving additional lectins, non-MBP carbohydrate-binding proteins, cytoskeletal or membrane-associated sensors, and other microbial-associated cues present on the bacterial surface. Nevertheless, this aligns with reports that soluble mannose analogues competitively mask mannose-sensitive lectins (Garate et al. 2005, Yoo and Jung 2012).

Overall, *Acanthamoeba* chemotaxis appears modular, with ligand-specific pathways tuned differently across the tested strains; *A. castellanii*’s broader sensitivity may reflect a generalist foraging strategy, whereas *A. polyphaga*’s selective fMLP bias suggests tighter receptor gating or downstream signalling tuned to specific microbial cues. These differences between the tested strains are consistent with fine-scale tuning of sensing thresholds, whereby ligand concentration and contextual signal load determine whether exploratory, directional, or suppressive behavioural programmes are engaged. Consistent with such a threshold-based sensing system, we additionally observed that exposure of *A. castellanii* to extremely high bacterial loads (MOI ∼1000) of our model bacterium triggered rapid morphological arrest and cessation of bacterial uptake, with cells adopting a rounded, cyst-like appearance within minutes. Additionally, cells remained morphologically intact without evidence of lysis during the observation period (Supplementary Fig. 2). Rather than indicating an active inhibitory signal, this response is most consistent with saturation or overwhelming of amoeba sensing and stress-response pathways under extreme microbial ligand burden, resulting in a switch from exploratory or phagocytic behaviour to acute protective arrest. At excessive bacterial densities, cumulative MAMP exposure may therefore exceed the operational range of amoeba sensing systems, reinforcing the concept that chemotactic and phagocytic behaviours in *Acanthamoeba* are governed by ligand-promiscuous, threshold-tuned pattern-recognition mechanisms rather than linear, high-affinity receptor engagement.

While both species remain valuable for comparative investigation, the broader chemotactic range of *A. castellanii* may provide a useful framework for probing general mechanisms of prey detection and microbe–protist interactions. While only a single strain per species was examined, these observations highlight strain-level variation that may reflect broader species-level divergence, which will require validation across additional isolates. Future comparative analyses will be essential to understand how recognition strategies vary across *Acanthamoeba*, and how evolutionary or ecological factors such as differences lectin repertoires shape this diversity.

### Genomic interrogation fails to identify a canonical formyl peptide receptor in *Acanthamoeba*

To determine whether chemotactic responses to N-formyl peptides are mediated by a canonical formyl peptide receptor (FPR), we interrogated the curated *Acanthamoeba castellanii* Neff genome (GCA_000313135.1) (Clarke et al. 2013) and the currently available *A. polyphaga* genome (GCA_000826345.1) (Chelkha et al. 2018). Proteomes were screened using HMMER (hmmscan) (Finn et al. 2011) against the Pfam-A database (Finn et al. 2013), focusing on GPCR families implicated in peptide sensing, including rhodopsin-like receptors (PF00001; Family 1), secretin-like receptors (PF00002; Family 2), Dictyostelid cAMP receptors (PF08395; Family 5), and Frizzled/Smoothened receptors (Family 6).

Candidate sequences were further assessed by BLASTP against the NCBI non-redundant database, predicted transmembrane topology, and inspection for conserved Class A GPCR hallmarks, including the DRY/DRH motif (TM3), CWxP motif (TM6), and NPxxY motif (TM7). Although several PF00001-annotated proteins were detected, high-scoring candidates (e.g. XP_004367874.1, XP_004345897.1, XP_004340808.1, XP_004368244.1) corresponded to kinases, adaptor proteins, RasGEFs, transporters, or RNA-associated proteins and lacked a coherent seven-transmembrane architecture. In these cases, predicted hydrophobic segments reflected internal helices or low-complexity regions rather than a convincing canonical GPCR fold. Across both genomes, all PF00001-annotated candidates identified by HMM-based screening were systematically evaluated based on transmembrane topology, conserved motif presence, and functional annotation. A full summary of all candidates, classification criteria, and exclusion rationale is provided in Supplementary File_4. While a subset of candidates exhibited multi-pass membrane topology consistent with GPCR-like architecture, none displayed a coherent combination of conserved Class A GPCR hallmarks, including appropriately positioned DRY/DRH, CWxP, and NPxxY motifs within a well-defined seven-transmembrane framework. In several cases, predicted helices were irregular or incomplete, and BLAST annotations indicated non-receptor functions such as kinases, transporters, or signalling adaptors.

Comparable analyses were performed on the currently available *A. polyphaga* genome (GCA_000826345.1) using the same HMMER-based screening and topology-filtering pipeline. While several sequences containing GPCR-associated domains were identified, none exhibited a coherent seven-transmembrane architecture with appropriately positioned Class A GPCR motifs or consistent receptor-like annotation. However, given the more limited annotation of the *A. polyphaga* genome, the absence of a convincing formyl peptide receptor ortholog remains to be confirmed. Importantly, we did not identify any canonical metazoan-like formyl peptide receptor orthologs in the analysed genomes. Canonical formyl peptide receptors are well characterised in metazoans, but their distribution outside animal lineages remains poorly defined, and there is currently no clear evidence supporting their conservation in Amoebozoa.

Given the reliance on reference assemblies and the absence of strain-resolved genomic data, these findings should be interpreted as an absence of convincing candidates under the applied search criteria rather than definitive evidence of receptor absence, despite robust behavioural responses to N-formyl peptides. This dissociation between chemotactic function and classical receptor homology supports a model in which peptide sensing may be mediated by, or arising from a divergent, pattern-recognition receptor–like system characterized by ligand promiscuity, potentially involving atypical GPCRs, non-GPCR membrane sensors, or lectin-associated signalling complexes that converge on conserved downstream pathways such as Ras-mediated chemotactic adaptation. Such an arrangement parallels emerging concepts in innate immunity, where conserved microbial ligands are detected by structurally diverse receptors that nevertheless drive analogous behavioural outcomes (Raabe et al. 2019).

### Formyl peptide modulation differs between *Acanthamoeba* species

**Figure 3:**
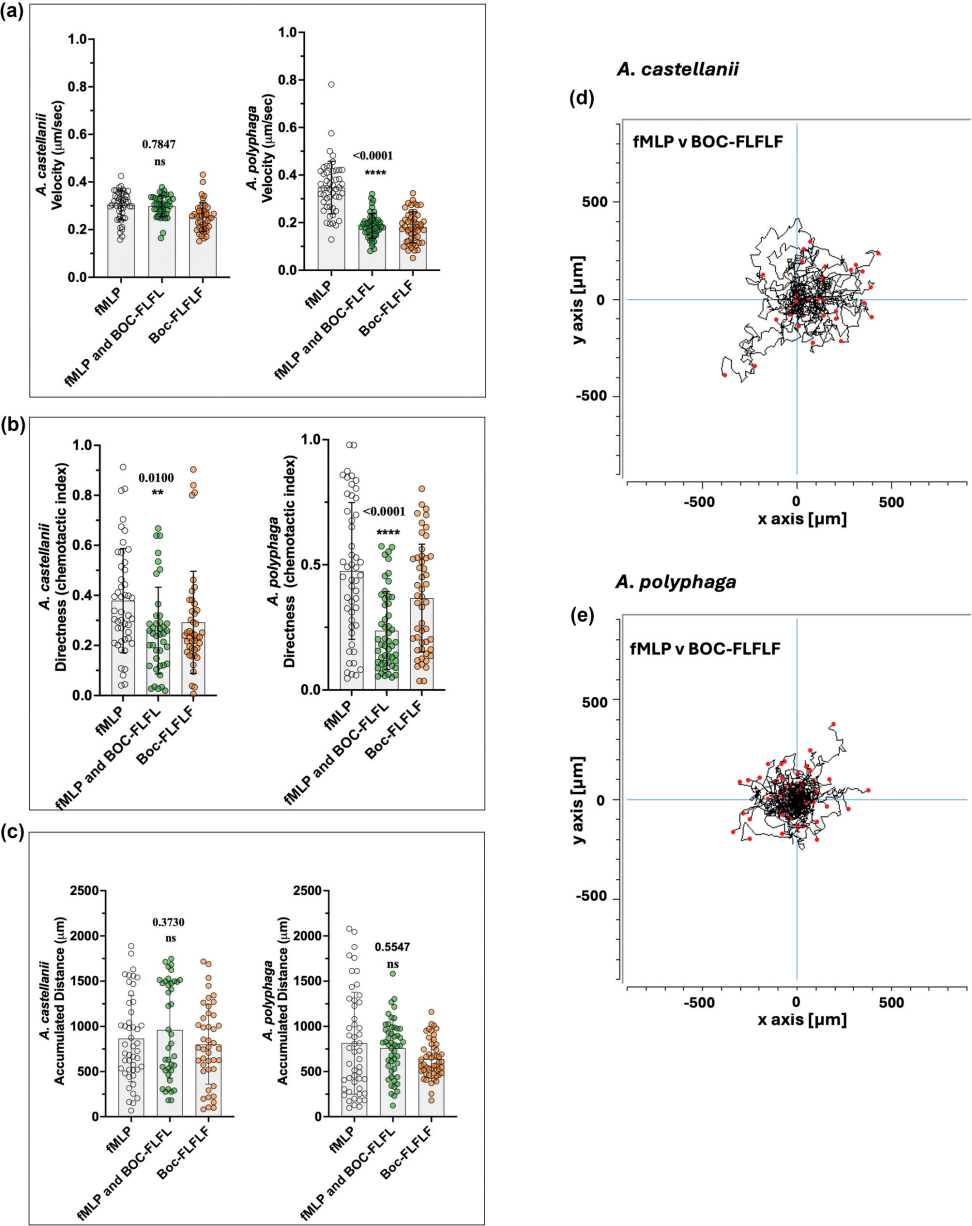
Effect of BOC-FLFLF on *A. castellanii* and *A. polyphaga* chemotaxis toward fMLP. *Acanthamoeba* responses were compared under three conditions: fMLP alone, fMLP + BOC-FLFLF and BOC-FLFLF alone. (a) Migration velocity (µm/min) between fMLP and fMLP + BOC-FLFLF. (b) Directness (chemotactic index) (c) accumulated distance travelled. Trajectory plot showing the effects of BOC-FLFLF on fMLP on (d) *A. castellanii* and (e) *A. polyphaga*. Data represent mean ± SD from three biological replicates (n = ∼45–55 cells/condition). Statistical comparisons were made using one-way ANOVA followed by Dunnett’s multiple comparisons test; significance levels: **P < 0.01. (Figure 3 was designed as a modulation assay comparing fMLP, BOC-FLFLF, and their combination within the same experimental context; baseline responses relative to ADM are shown in Figs. 1 and 2).

We sought to determine whether *Acanthamoeba* species exhibit mammalian-like formyl peptide modulation, particularly whether chemotactic responses to fMLP are antagonised by BOC-FLFLF, a known competitive inhibitor of the mammalian formyl peptide receptor (FPR). To test this, we exposed *A. castellanii* and *A. polyphaga* to fMLP alone, BOC-FLFLF alone, and a combination of both (Fig. 3).

In neutrophils, fMLP is a potent chemotactic agonist, while BOC-FLFLF is a selective FPR antagonist that blocks fMLP-driven signalling (Johansson et al. 1993, Hayashi et al. 2014).

In *A. castellanii*, the addition of BOC-FLFLF to fMLP did not significantly (*P* > 0.05) affect velocity or accumulated distance relative to fMLP alone, but both parameters remained comparable to BOC-FLFLF alone. However, directness was significantly reduced (*p*<0.01) compared to fMLP alone and matched the reduced directionality seen with BOC-FLFLF alone. These findings suggest that while BOC-FLFLF suppresses directional sensing in *A. castellanii*, it does not interfere with overall motility or path length. In contrast, *A. polyphaga* exhibited a broader suppressive response: the fMLP + BOC-FLFLF combination significantly reduced both velocity (*P* < 0.0001) and directness (*P* < 0.0001) relative to fMLP alone, while these reductions were not significant compared to BOC-FLFLF alone. Accumulated distance remained unchanged across conditions. This indicates that in *A. polyphaga*, BOC-FLFLF more broadly dampens chemotactic responses, reducing both speed and orientation, potentially reflecting lower signalling thresholds or less selective peptide sensing mechanisms (Fig. 3a–c). Trajectory plots further support these distinctions, revealing diminished and disoriented migration tracks in both species under co-treatment, but with more profound motility suppression in *A. polyphaga* (Fig. 3d–e). It is worth noting that although trajectory plots in *A. castellanii* suggest subtle differences between fMLP and BOC-FLFLF conditions, these did not reach statistical significance across quantitative metrics, reflecting high variability and a relatively modest effect size. In contrast, *A. polyphaga* exhibited a more pronounced and statistically significant inhibitory response to BOC-FLFLF.

These differences between the tested strains, consistent with species-level divergence, suggest that formyl peptide recognition and antagonism are modulated by divergent receptor sensitivities or signalling architectures. The ability of *A. castellanii* to sustain motility while selectively losing directional bias may indicate the presence of a more finely tuned sensing system. These behavioural outcomes, particularly the selective suppression of directness in *A. castellanii* without impairment of motility, are reminiscent of GPCR-regulated chemotactic adaptation observed in neutrophils and *Dictyostelium* systems. Importantly, the absence of a canonical formyl peptide receptor homolog does not preclude GPCR-mediated peptide sensing in *Acanthamoeba* but instead indicates that peptide detection is likely mediated by a structurally divergent receptor architecture that converges on conserved Ras-based chemotactic signalling. In human neutrophils, CAPRI (a calcium-promoted Ras inactivator) is essential to regulate GPCR-mediated Ras activation and adaptation, thereby controlling cell sensitivity across a wide range of chemoattractant concentrations. CAPRI also fine-tunes directional sensing by locally deactivating Ras following fMLP stimulation (Xu et al. 2021, 2021). The broader suppression of both velocity and directionality in *A. polyphaga* upon co-treatment with BOC-FLFLF may reflect species-specific differences in receptor signalling strength, ligand binding affinity, or the responsiveness of downstream Ras regulatory networks. Although canonical FPR homologs remain unidentified in *Acanthamoeba*, the functional parallels we observe, particularly the selective antagonism of directional sensing, strongly suggest the existence of a divergent yet functionally analogous GPCR–Ras signalling circuit mediating peptide-driven chemotaxis. Indeed, genomic analysis reveals that *A. castellanii* encodes at least 35 putative GPCRs across several classes (families 1, 2, and 6) and five G-protein α-subunit genes, implying a sophisticated signalling network capable of discriminating peptide-based cues (Clarke et al. 2013). Genomic context supports a mechanistic basis for divergence: *A. castellanii* (Neff) harbours multiple GPCR families and lectins in a curated genome, whereas *A. polyphaga* currently has a larger, draft nuclear assembly with less complete receptor annotation. Together with species-specific microbial associations (e.g. giant virus interactions in *A. polyphaga*) (Chelkha et al. 2018), these differences plausibly tune receptor repertoires and signalling thresholds, aligning with the broader *A. castellanii* and more selective *A. polyphaga* chemotactic signatures we observed.

## Conclusion

By revisiting and expanding upon the foundational chemotaxis work by Schuster and Levandowsky (Schuster and Levandowsky 1996a) with microfluidic gradients and frame-by-frame tracking, we validate most of this classic study. *Acanthamoeba castellanii* migrates strongly to mannan, fMLP and GlcNAc and shows little response to MurNAc or BOC-FLFLF but revealed three new layers of insight. First, stable nano-litre gradients uncovered clear attraction to mannan/mannose that the earlier millimetre-scale slide could not resolve, consistent with the species’ mannose-binding–proteins (Corsaro 2022). Second, comparative analysis reveals that *A. castellanii* exhibits a broader and more nuanced chemotactic repertoire than *A. polyphaga*, with stronger responses to multiple glycans and peptides with a more robust directional sensing. In contrast to *A. polyphaga* which displayed a narrower chemotactic profile, characterized by selective sensitivity to fMLP and broader suppression in response to BOC-FLFLF. Third, these behavioural contrasts are compatible with GPCR-Ras adaptation paradigms borrowed from neutrophils and *Dictyostelium*, where Ras-GAP tuning can uncouple velocity from directional bias (Duda-Chodak et al. 2015), although the underlying receptors in *Acanthamoeba* remain unidentified.

Although both species are frequently isolated from the same environmental sources, including soil, freshwater systems, drinking water, air, and biofilms, with no published evidence supporting distinct niche partitioning, we hypothesise that their divergent chemotactic “signatures” reflect micro-niche specialisation, fine-tuning of receptor repertoires to local prey spectra rather than habitat partitioning. Our data establish quantitative benchmarks that can guide subsequent efforts to link ligand classes to specific *Acanthamoeba* receptors, pending targeted genetic and pharmacological validation.

## Supplementary Material

uqag023_Supplemental_Files

## Data Availability

All data generated or analysed during this study are included in the manuscript and supporting files.
