# Supplementary material for: Divergent chemotactic sensing in Acanthamoeba reveals ligand-promiscuous, threshold-tuned pattern recognition without canonical formyl peptide receptors
Source: Microlife. 2026 Jun 27;7:uqag023. doi: 10.1093/femsml/uqag023 (PMC13359236; doi:10.1093/femsml/uqag023)
Supplement: uqag023_Supplemental_Files [file uqag023_supplemental_files.zip › Supplementary File 3.docx]

**Divergent Chemotactic Sensing in Acanthamoeba: Evidence for Ligand-Promiscuous, Threshold-Tuned Pattern Recognition in the Absence of Canonical Formyl Peptide Receptors**

Viktor Hermaraj^1^, Brendan W. Wren^1^, Fauzy Nasher^1*^

^1^Department of Infection Biology

London School of Hygiene and Tropical Medicine

Keppel St, London WC1E 7HT, United Kingdom.

***Correspondence:** Fauzy Nasher ([fauzy.nasher1@lshtm.ac.uk](mailto:fauzy.nasher1@lshtm.ac.uk))


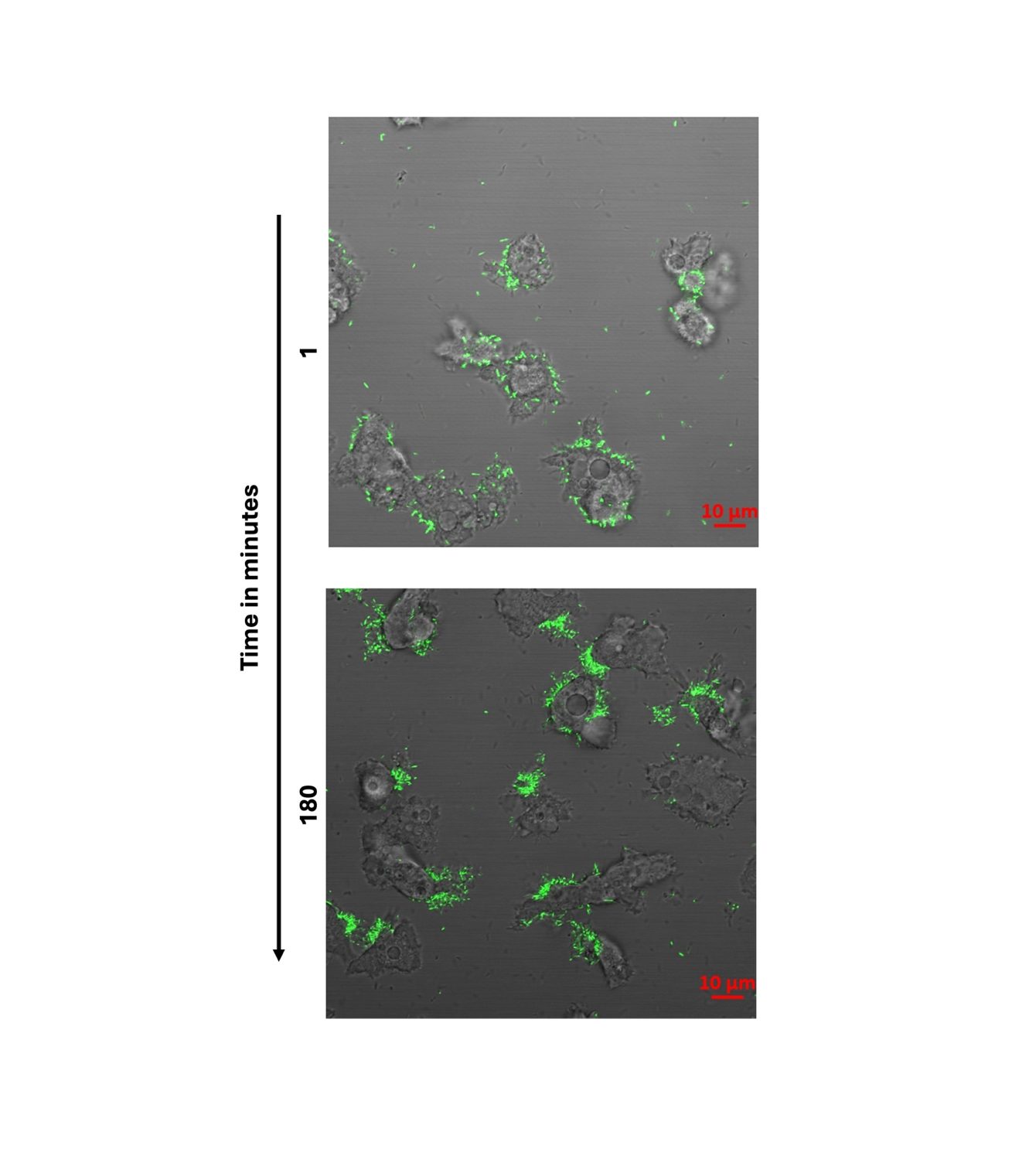


**Supplementary Figure 1: Mannose pre-exposure promotes surface accumulation of bacteria on the surface Acanthamoeba castellanii.** **a)** Trophozoites were pre-exposed to 1mM of **mannose** for 30 min in ADM, then infected with **GFP-expressing** C. jejuni at **MOI =** ~**100**. Interactions were imaged live in ADM for ~3 h (time lapse images were acquired at 5 seconds intervals). Following mannose pre-exposure, bacteria accumulate along the amoeba surface without internalization over time, consistent with **adhesion-without-uptake** phenotype under these conditions. **Zeiss LSM880** Differential interference contrast (DIC) (grey), overlaid with GFP fluorescence (green). Representative fields are shown at **t = 1 min** (top) and **t = 180 min** (bottom). **Scale bar: 10 µm.** (Baseline uptake in the absence of mannose pre-exposure is shown in Supplementary Figure 2b)**.**

**b)** Intracellular bacteria were quantified by gentamicin protection assays and expressed as CFU/mL. Infections were performed at an MOI = ~100Mannose pre-exposure resulted in increased bacterial association prior to gentamicin treatment (Pre-gen +mannose) (3 hr p.i), consistent with surface adhesion. However, no recoverable intracellular bacteria were detected following gentamicin treatment (Post-gen +mannose; indicated by arrow showing no detectable CFU) (4 hr p.i; including 1h gentamicin treatment), indicating inhibition of productive uptake. Baseline uptake in the absence of mannose pre-exposure is shown alongside (Post-gen -mannose). * = *P*<0.05; data is presented as Log_10_ transformed; standard deviation and each point represents a biological replicate (three biological replicates were performed).

**
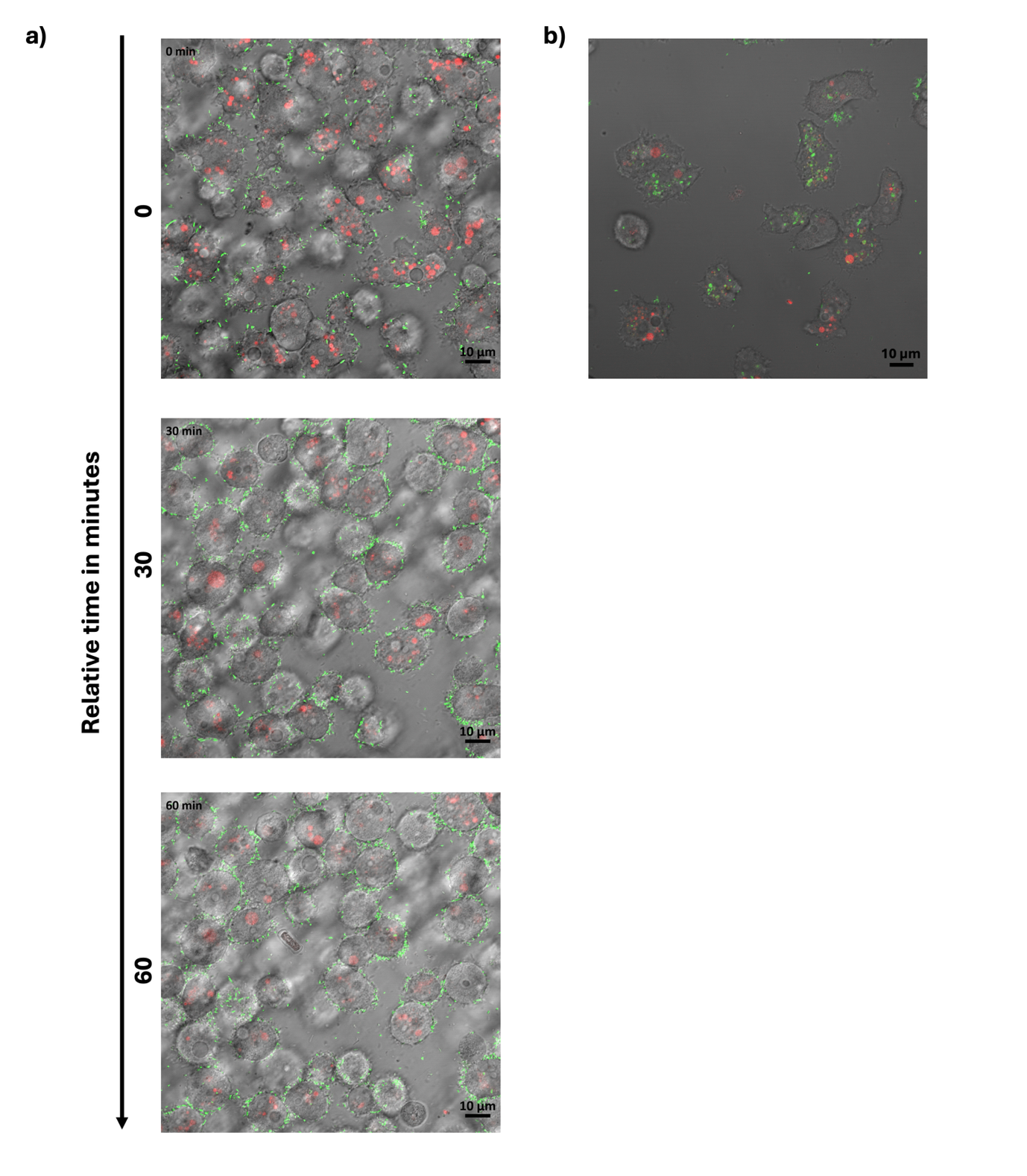
**

**Supplementary Figure 2. a)** Exposure of *A. castellanii* to *C. jejuni* at MOI = ~1000 resulted in rapid rounding and adoption of a cyst-like morphology, accompanied by cessation of observable bacterial uptake and motility. This response occurred within minutes of exposure and was not associated with progressive lysis, consistent with an acute behavioural shutdown rather than delayed cytotoxicity. **b)** Lower bacterial loads (MOI = ~100) supported active motility and uptake. These observations are consistent with a threshold-based suppression of exploratory behaviour under overwhelming ligand input.

All experiments presented in Supplementary File 3 were performed in accordance with previously published methods, including the use of *Campylobacter jejuni*_GFP_ expressing strain (Nasher et al., 2022; Nasher et al., 2025).

**Reference:**

Nasher, F., Lehri, B., Horney, M.F., Stabler, R.A., and Wren, B.W. (2022). Survival of Campylobacter jejuni 11168H in Acanthamoebae castellanii Provides Mechanistic Insight into Host Pathogen Interactions. *Microorganisms* 10(10). doi: 10.3390/microorganisms10101894.

Nasher, F., Lehri, B., Stabler, R.A., and Wren, B.W. (2025). Acanthamoeba castellanii as a model for unveiling Campylobacter jejuni host–pathogen dynamics. *Frontiers in Cellular and Infection Microbiology* Volume 15 - 2025. doi: 10.3389/fcimb.2025.1583830.
